# Systemic lupus erythematosus variants modulate the function of an enhancer upstream of *TNFAIP3*

**DOI:** 10.3389/fgene.2022.1011965

**Published:** 2022-09-19

**Authors:** Satish Pasula, Jaanam Gopalakrishnan, Yao Fu, Kandice L. Tessneer, Mandi M. Wiley, Richard C. Pelikan, Jennifer A. Kelly, Patrick M. Gaffney

**Affiliations:** ^1^ Genes and Human Disease Research Program, Oklahoma Medical Research Foundation, Oklahoma City, OK, United States; ^2^ Department of Pathology, University of Oklahoma Health Sciences Center, Oklahoma City, OK, United States

**Keywords:** systemic lupus erythematosus, TNFAIP3, IL20RA, IFNGR1, functional genetics

## Abstract

*TNFAIP3*/A20 is a prominent autoimmune disease risk locus that is correlated with hypomorphic *TNFAIP3* expression and exhibits complex chromatin architecture with over 30 predicted enhancers. This study aimed to functionally characterize an enhancer ∼55 kb upstream of the *TNFAIP3* promoter marked by the systemic lupus erythematosus (SLE) risk haplotype index SNP, rs10499197. Allele effects of rs10499197, rs58905141, and rs9494868 were tested by EMSA and/or luciferase reporter assays in immune cell types. Co-immunoprecipitation, ChIP-qPCR, and 3C-qPCR were performed on patient-derived EBV B cells homozygous for the non-risk or SLE risk *TNFAIP3* haplotype to assess haplotype-specific effects on transcription factor binding and chromatin regulation at the *TNFAIP3* locus. This study found that the *TNFAIP3* locus has a complex chromatin regulatory network that spans ∼1M bp from the promoter region of *IL20RA* to the 3′ untranslated region of *TNFAIP3*. Functional dissection of the enhancer demonstrated co-dependency of the RelA/p65 and CEBPB binding motifs that, together, increase *IL20RA* and *IFNGR1* expression and decreased *TNFAIP3* expression in the context of the *TNFAIP3* SLE risk haplotype through dynamic long-range interactions up- and downstream. Examination of SNPs in linkage disequilibrium (*D’* = 1.0) with rs10499197 identified rs9494868 as a functional SNP with risk allele-specific increase in nuclear factor binding and enhancer activation *in vitro*. In summary, this study demonstrates that SNPs carried on the ∼109 kb SLE risk haplotype facilitate hypermorphic *IL20RA* and *IFNGR1* expression, while suppressing *TNFAIP3* expression, adding to the mechanistic potency of this critically important locus in autoimmune disease pathology.

## Introduction

Tumor necrosis factor alpha-inducible protein 3 (*TNFAIP3*) on chromosome 6q23 encodes the ubiquitin-modifying enzyme, A20 (Ma and Malynn, 2012; Das et al., 2018). *TNFAIP3*/A20 expression is induced in response to pro-inflammatory stimuli, serves as a negative feedback mechanism that attenuates nuclear factor kappa-Β (NFκB), toll-like receptor, and nucleotide-binding oligomerization domain signaling, and protects against the effects of chronic inflammation. Global and immune cell-specific A20 deficiency in animal models increases pro-inflammatory cytokine and chemokine production, chronic inflammation, immune cell dysfunctions, and loss of self-tolerance consistent with autoimmune phenotypes (Das et al., 2018). Haploinsufficiency of *TNFAIP3* also leads to a monogenic autoinflammatory disorder in humans (Zhou et al., 2015; Franco-Jarava et al., 2018; Tsuchida et al., 2019). Genome-wide association studies (GWAS) have associated the *TNFAIP3* locus with at least 16 different human diseases including systemic lupus erythematosus (SLE), rheumatoid arthritis, Sjögren’s disease, systemic sclerosis, and psoriasis (Plenge et al., 2007; Thomson et al., 2007; Graham et al., 2008; Bates et al., 2009; Han et al., 2009; Nair et al., 2009; Trynka et al., 2009; Cai et al., 2010; Dieudé et al., 2010; Shimane et al., 2010; Strange et al., 2010; Adrianto et al., 2011; Musone et al., 2011; Koumakis et al., 2012; Xu et al., 2019; Ray et al., 2020; Khatri et al., 2022).

*TNFAIP3* is in an ∼300 kb topologically associating domain (TAD) that exhibits complex chromatin architecture including two topologically associating subdomains (sub-TADs) and more than 30 predicted enhancers with varying epigenetic activity (Sokhi et al., 2018; Ray et al., 2020). Spanning this TAD are 15 disease-associated haplotypes from European or East Asian ancestry, including the ∼109 kb SLE risk haplotype correlated with reduced *TNFAIP3* gene expression in SLE patient populations of European and Korean ancestry (Graham et al., 2008; Adrianto et al., 2011; Ray et al., 2020). Several SNPs carried on this SLE risk haplotype are non-protein-coding SNPs shared by other *TNFAIP3* haplotypes and positioned in predicted enhancer elements based on chromatin accessibility, histone marks of active enhancers, and enrichment of transcription factor binding sites (Sokhi et al., 2018; Ray et al., 2020). The TT>A enhancer positioned ∼42 kb downstream of the *TNFAIP3* gene body is arguably one of the most well characterized. The TT>A enhancer facilitates *TNFAIP3* gene expression by binding and delivering NFκΒ to the *TNFAIP3* promoter via SATB1-mediated long-range DNA looping (Wang et al., 2013; Wang et al., 2016). The TT>A risk alleles for SLE (rs148314165 T/-, rs200820567 T>A) impaired NFκΒ binding and DNA looping thus disrupting transcription factor delivery and reducing *TNFAIP3* expression. Given that a single gene promoter has the potential to simultaneously engage and be regulated by multiple distant enhancers and, alternatively, that enhancers can modulate different genes in different cell types and contexts, determining how risk alleles disrupt gene regulation through modulating enhancer function in the context of specific cell types and environmental contexts is critical for understanding autoimmune disease pathology.

In this study, we functionally characterized an enhancer ∼55 kb upstream of *TNFAIP3* with two SNPs, rs58905141 and rs10499197, that have strong bioinformatic evidence of functionality and are shared by the ∼109 kb SLE risk haplotype and others (Ray et al., 2020). Functional dissection of this enhancer demonstrated that it forms long-range contacts with several regulatory regions, including an interaction with the upstream promoter regions of *IL20RA* and *IFNGR1*. The long-range interaction between this enhancer and *IL20RA* and *IFNGR1* in Epstein-Barr virus (EBV) transformed B (EBV B) cells from donors carrying the *TNFAIP3* SLE risk haplotype tagged by rs10499197 exhibited increased interaction frequency, as well as increased *IL20RA* and *IFNGR1* expression, suggesting the risk haplotype may influence gene expression beyond *TNFAIP3*.

## Materials and methods

### Antibodies and cell lines

Jurkat and THP-1 cells were purchased from ATCC. EBV B cell lines were derived from SLE patients enrolled into the Lupus Family Registry and Repository (LFRR) and obtained from the Oklahoma Medical Research Foundation’s (OMRF) Arthritis and Clinical Immunology Biorepository Core with IRB approval (Rasmussen et al., 2011). Genotypes of EBV B cell lines carrying the non-risk or risk alleles of rs10499197, rs58905141, or rs9494868 were verified by Sanger sequencing. Cell lines were maintained in RPMI 1640 medium supplemented with 10% FBS, 1X penicillin-streptomycin antibiotic mixture (Atlanta Biologicals Inc.), and 2 mML-glutamine (Lonza). THP-1 cell medium was supplemented with 55 μM ß-mercaptoethanol. Cells were stimulated with or without 50 ng/ml phorbol 12-myristate 13-acetate and 500 ng/ml ionomycin (P/I) for 2 h prior to harvest. All stock laboratory chemicals were from Sigma Aldrich or ThermoFischer.

### HiChIP

Histone H3 Lysine 27 acetylation (H3K27ac)-mediated chromatin interactions in EBV B cells were previously published [NCBI GEO (https://www.ncbi.nlm.nih.gov/geo/): GSE116193] (Pelikan et al., 2018). HiChIP FASTQ files were aligned to the hg19 human reference genome using HiC-Pro (http://github.com/nservant/HiC-Pro) (Servant et al., 2015), then processed and analyzed through the hichipper pipeline (https://github.com/aryeelab/hichipper) (Lareau and Aryee, 2018). MAC2 was used for anchor calling based on ChIP-enriched regions (Zhang et al., 2008). Loops were derived from the linked paired-end reads that overlap anchors, then visualized in two dimensions using DNAlandscapeR (https://github.com/aryeelab/DNAlandscapeR).

### Radiolabeled electrophoretic mobility shift and supershift assays

Complementary pairs of ∼40bp non-risk and risk probes were chemically synthesized by Integrated DNA Technologies, annealed, and end-labeled with (γ-^32^P) adenosine triphosphate (Perkin Elmer) using T4 polynucleotide kinase (New England Biolabs (NEB); #M0291S) (Supplementary Table S1). Ten micrograms of nuclear proteins, extracted from Jurkat, EBV B, and THP-1 cells stimulated with or without P/I, were incubated with labelled 50,000 cpm probes in binding buffer (1 μg poly dI-dC, 20 mM HEPES, 1mM MgCl_2_, 100 mM Tris-HCl, pH 7.4, and 0.5 mM EDTA) for 30min at room temperature. DNA-protein complexes were resolved on non-denaturing 5% acrylamide gels in 0.5X Tris borate/EDTA. Dried gels were exposed overnight on a phosphor screen, visualized on a phophor-imager (BioRad; GS-360), and quantified using BioRad Quantity 1D Analysis software. For competition assays, 10, 50, and 100-fold excess of unlabeled non-risk and risk probes were added to the binding reactions. For the EMSA-SS, anti-p65 antibody (GeneTex, Inc.; #GTX107678) was added to the binding reaction and incubated for 15 min at room temperature prior to adding labeled probe.

### Dual luciferase assay

Non-risk or risk sequences surrounding rs10499197 or rs58905141 were cloned from EBV B cells positive for the non-risk or risk *TNFAIP3* SLE risk haplotype tagged by rs10499197 into a minimal promoter firefly luciferase plasmid, pGL4.23 (Promega) (Supplementary Table S2) (Wang et al., 2013; Pasula et al., 2020; Gopalakrishnan et al., 2022). Quick change II site directed mutagenesis kit (Agilent Technologies; #200523) was used following manufacturer’s instructions to allele swap rs9494868 (Supplementary Table S2). Empty vector, non-risk clone, or risk clone was transiently co-transfected with the transfection control renilla luciferase plasmid, pRL-TK, using a 4D Amaxa Nucleofector Unit (Lonza) for Jurkat cells (Nucleofector SE kit; #V4XC-1032), THP-1 cells (Nucleofector SG kit; #V4XC-3032), and EBV B cells (Nucleofector SF kit; #V4XC-2032). Twenty-four hours post transfection, cells were stimulated with P/I for 2 h. Enhancer activity was determined using the Promega Dual-Luciferase Reporter Assay kit (Promega) following manufacturer’s protocol. Relative luciferase units (RLU) were determined by normalizing the firefly luciferase activity to the Renilla luciferase activity. RLU were normalized to the vector-only control and reported as normalized RLU.

### Chromatin conformation capture with quantitative PCR (3C-qPCR)

Ten million cells were collected from EBV B cell lines: three homozygous risk and three homozygous non-risk for the rs10499197-tagged *TNFAIP3* SLE risk haplotype, and three homozygous risk for rs9494868 on the genetic background of homozygous non-risk for the rs10499197-tagged *TNFAIP3* SLE risk haplotype. Cells were crosslinked with 2% formaldehyde at room temperature for 10min, then quenched with 125 mM glycine for 5min at room temperature. Cells were lysed, then nuclei were pelleted and resuspended in 1.25X CutSmart Buffer (NEB; #B7204S). Nuclei were treated with 0.3% SDS at 37°C for 1 h, then 2% Triton X-100 for 1 h. Chromatin was digested overnight at 37°C using 5000U HindIII (NEB; #R3104M). Ligation was performed overnight at 16°C or on ice using T4 ligase (NEB; #M0202M), yielding similar results. Chromatin were de-crosslinked by incubating with Proteinase K overnight at 65°C. RNase A treatment removed RNA contamination. 3C DNA template was purified twice by phenol:chloroform:isoamyl alcohol extraction. 3C-qPCR primers were designed to anneal in close proximity to HindIII cutting sites and amplify seven fragments spanning either the downstream *TNFAIP3* locus or the upstream promoter regions of *IL20RA* and *IFNGR1* (Supplementary Table S3). The anchor fragment contained rs58905141 and rs10499197. Primer efficiency was validated and normalized using template DNA from BAC clones (Thermofischer), RP11-16209, RP11-1058B18, CTD-2071P12, CTD-3175H21, RP11-790N23 mixed in equimolar ratios and digested with HindIII.

All qPCR assays were performed using LightCycler480 SYBR Green probe according to manufacturer’s instructions (Roche Diagnostics). Relative interaction frequencies (RIFs) were calculated by normalizing the interaction frequencies to the interaction frequency of the random ligation control (RLC) (Hagege et al., 2007). Positive interactions had a RIF greater than the interactions between the anchor and the negative control region (NCR).

### RNA extraction and quantitative reverse transcription-PCR (qRT-PCR)

Total RNA was isolated from EBV B cells carrying the rs10499197-tagged *TNFAIP3* non-risk or risk haplotype using Direct-zol RNA MiniPrep Plus kit (Zymo Research) according to the manufacturer’s protocol. cDNA synthesis was performed using QuantiTect reverse transcriptase kit (Qiagen) as per the manufacturer’s recommendations. Gene expression was measured by qRT-PCR analysis using Light Cycler480 SYBR Green (Roche Diagnostics). Gene expression primers for human *IL20RA* (QT00069272), *IFNGR1* (QT00089404) and *GAPDH* (PPH00150F) were purchased from Qiagen.

### Immunoprecipitation and western blot analyses

EBV B cells homozygous for the rs10499197-tagged non-risk *TNFAIP3* haplotype were collected, washed twice with 1X PBS and lysed with ice cold RIPA Buffer (1% Triton X-100, 0.1% SDS, 0.5% sodium deoxycholic acid, 5 mM tetrasodium pyrophosphate, 50 mM sodium fluoride, 5 mM EDTA, 150 mM NaCl, 25 mM Tris, pH 7.5, 5 mM Na_3_VO_4_, 5 mM N-ethylmaleimide, and protease inhibitor cocktail). Cell lysates were precleared with normal rabbit IgG (Cell Signaling, Inc.; #2729S) and protein A/G beads for 2 h at 4°C followed by incubation with anti-RelA/p65 antibody (GeneTex, Inc.; #GTX107678) for 16 h at 4°C. Precipitated proteins were eluted from beads using 2X Lamaelli buffer, then denatured at 95°C for 5min. Denatured proteins were separated by SDS-PAGE, transferred to polyvinylidene difluoride membrane (BioRad; #1620177), blocked with 5% non-fat dairy milk, and analyzed by western blotting using anti-RelA/p65 antibody (Sigma-Aldrich; #17-10060) or anti-CEBPB antibody (Santa Cruzy Biotechnology; #sc-7962). Proteins were detected using Pierce ECL Western Blotting Substrate (Thermo Fisher Scientific; #32106) and visualized using a ChemiDocMP Imaging System (Bio-Rad).

### Chromatin immunoprecipitation and quantitative PCR (ChIP-qPCR)

1 × 10^7^ EBV B cells homozygous for the *TNFAIP3* non-risk or risk haplotype were treated with P/I in growth medium for 2h, then cross-linked with 1% formaldehyde. TruChIP chromatin shearing kit (Covaris; #520154) was used to isolate and sonicate (Covaris S1 sonicator (#E220)) nuclei in 1 ml of lysis buffer according to manufacturer’s instructions. Sheared chromatin was precleared by incubating with Magna ChIP protein A/G beads for 1 h at 4 °C with mild agitation. Five hundred microliters of chromatin-protein complexes were immunoprecipitated overnight at 4 °C by mild agitation with Magna ChIP protein A/G beads (MilliporeSigma) and antibodies specific for RelA/p65 (Sigma Aldrich; #17–10060), CEBPB (Abcam; ab#32358 or Santa Cruz Biotechnology; #sc-7962), or normal rabbit IgG (negative control; Cell Signaling, Inc.; #2729S). DNA was eluted from the immunoprecipitated chromatin complexes, reverse-crosslinked, purified by Agencourt AMPure XP beads (Beckman Coulter), and subjected to real-time qPCR analysis using primers that flanked CEBPB or RelA/p65 binding motif in proximity to rs10499197 (Supplementary Table S4).

## Results

### Risk alleles of rs58905141 and rs10499197 alter the binding affinities of nuclear protein complexes to an enhancer ∼55 kb upstream of *TNFAIP3*

SNP rs10499197 is positioned ∼55 kb upstream of the *TNFAIP3* promoter in an enhancer element defined by H3K27ac enrichment and ChIP-seq transcription factor binding motifs (Supplementary Figure S1) (Tyner et al., 2017). SNP rs58905141 is positioned 393 bp upstream of and in linkage disequilibrium (LD) with rs10499197 (*r* ^*2*^ = 1.0; *D’* = 1.0) (Ensembl’s LD calculator: https://uswest.ensembl.org/Homo_sapiens/Tools/LD) (Supplementary Figure S1). HiChIP analysis in EBV B cells revealed a H3K27ac-mediated chromatin-chromatin interaction between this enhancer and a region spanning the *TNFAIP3* gene body (Supplementary Figure S1) (Pelikan et al., 2018), as well as to the previously characterized downstream TT>A enhancer (Supplementary Figure S1) (Wang et al., 2013; Wang et al., 2016; Sokhi et al., 2018).

**FIGURE 1:**
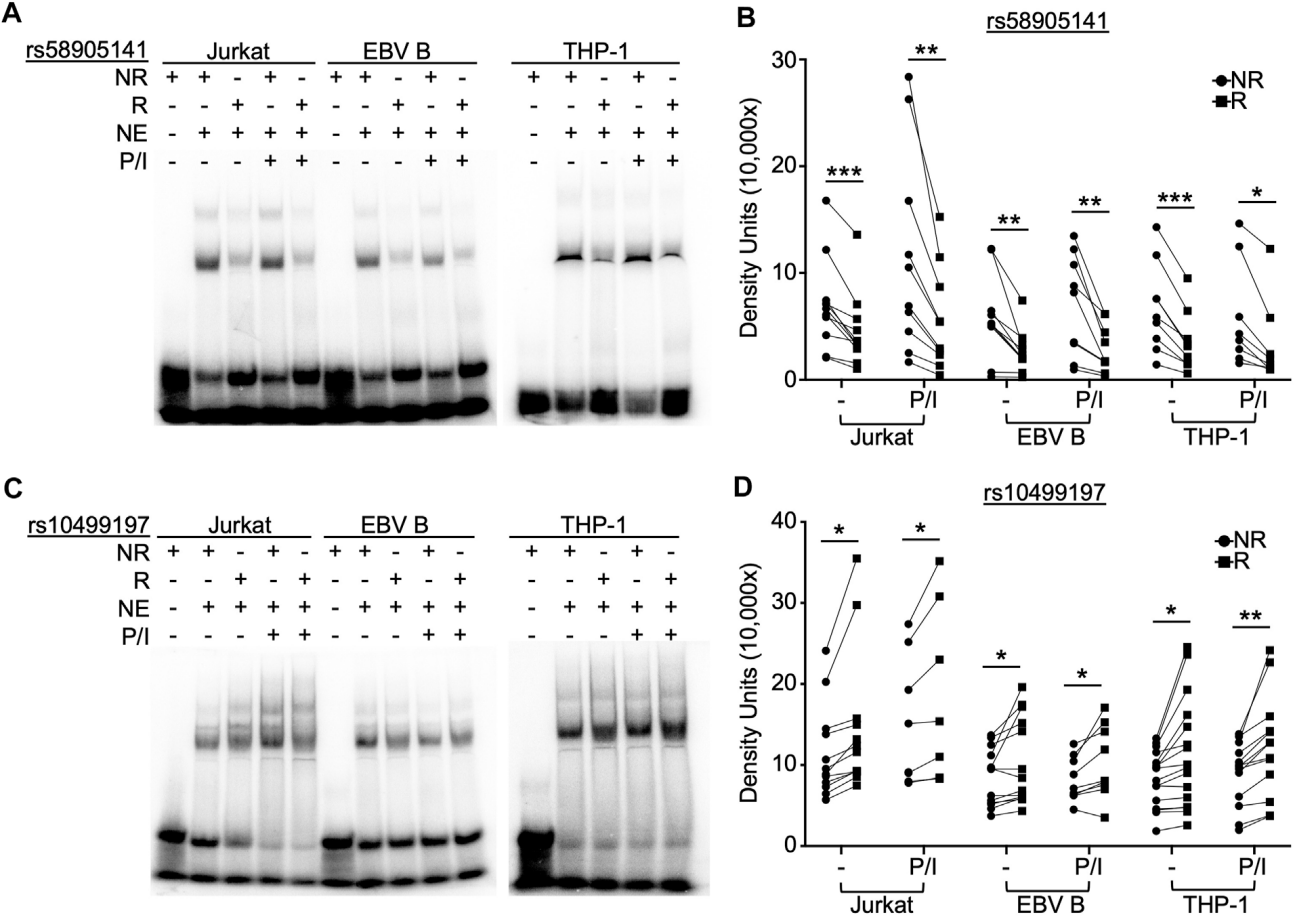
SNP rs58905141 and rs10499197 demonstrate allele-specific nuclear protein binding. EMSA using nuclear extracts from Jurkat, EBV B, and THP-1 cells stimulated with or without PMA/Ionomycin (P/I) for 2 h. Radiolabeled probes of **(A,B)** the non-risk (A) or risk (G) allele of rs58905141 or **(C,D)** the non-risk (T) or risk (G) allele of rs10499197 were incubated with nuclear extracts as indicated. **(A,C)** Images are representative of n > 8. **(B,D)** Densitometry was used to quantify nuclear factor-bound DNA probe; paired *t*-test: *p < 0.05, **p < 0.005; ***p < 0.001. (Figure Abbreviations: NE, nuclear extract; NR, non-risk allele; R, risk allele).

To examine the function of rs58905141 (A/G) and rs10499197 (T/G) on nuclear protein complex binding affinities, we performed radiolabeled EMSAs using nuclear extracts from Jurkat, EBV B, and THP-1 cells stimulated without or with P/I for 2 h. EMSA probe specificity was confirmed by competition binding with unlabeled probes in THP-1 cells (Supplementary Figure S2). The risk allele of rs58905141 significantly reduced nuclear protein complex binding compared to the non-risk allele across all cell types with or without P/I stimulation (Figures 1A,B, Supplementary Figure S3A). In contrast, the risk allele of rs10499197 exhibited an allele-specific change in band width distribution and significantly increased nuclear protein complex binding compared to the non-risk allele across all cell types and irrespective of stimulation (Figures 1C,D, Supplementary Figure S3B).

### The rs10499197 risk allele increases enhancer activity in immune cell lines

We used a dual luciferase expression system designed to examine the enhancer’s functionality and the potential allelic effects of rs58905141 and rs10499197. Sequences spanning the rs58905141 or rs10499197 variants were PCR-amplified using genomic DNA extracted from EBV B cells that were homozygous for the non-risk or risk variants, cloned into a minimal promoter luciferase expression vector, and transiently expressed in Jurkat, EBV B, or THP-1 cells. After 24 h and prior to measuring luciferase activity, cells were stimulated for 2 h without or with P/I. After normalization, increased luciferase activity over the vector-only control was interpreted as enhancer activation.

**FIGURE 2:**
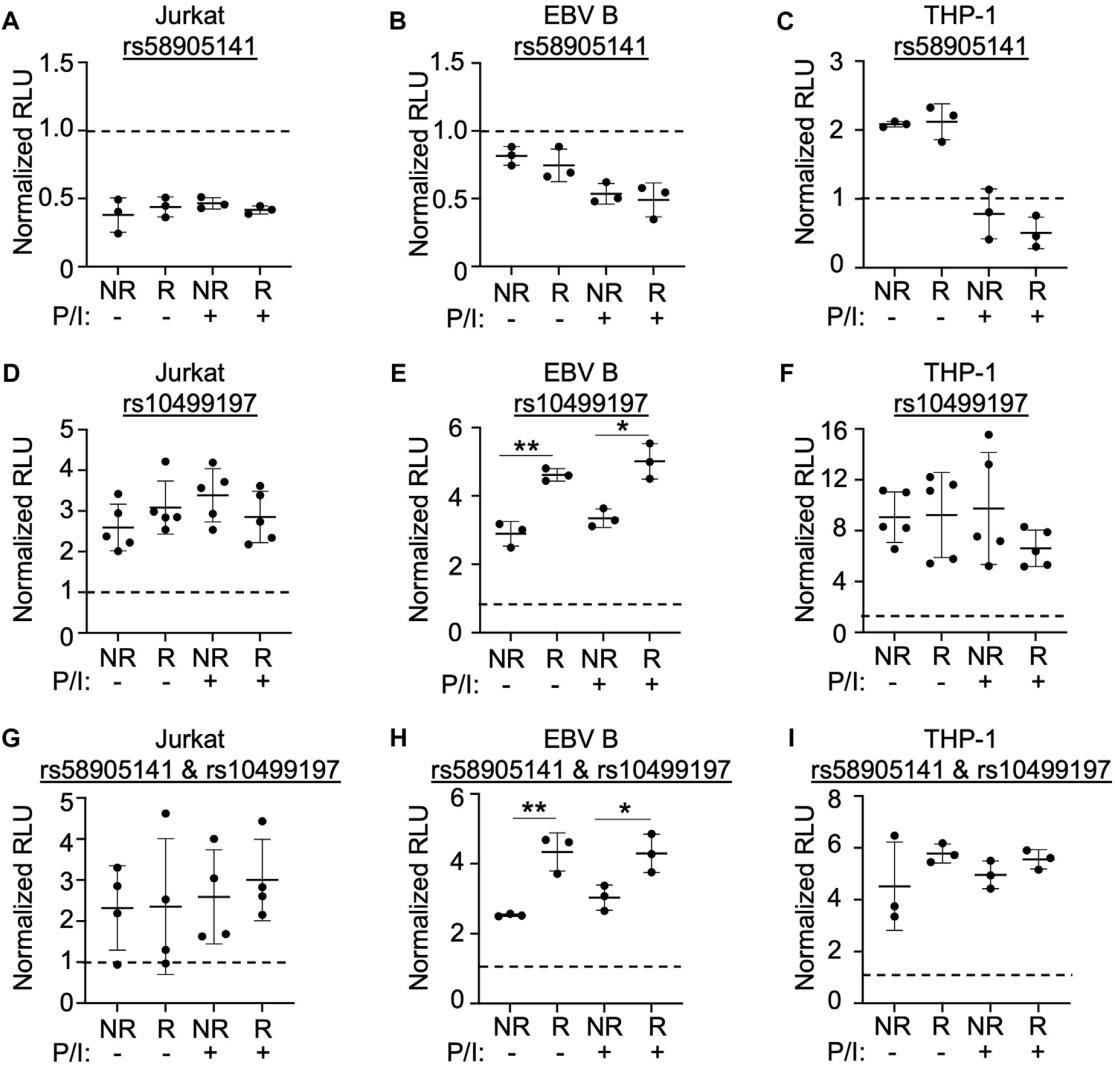
SNP rs58905141 and rs10499197 reveal novel enhancer function**. (A–F)** Dual luciferase assay using Jurkat **(A,D)**, EBV B **(B,E)**, or THP-1 **(C,F)** cells transiently transfected with empty pGL4.23, pGL4.23 containing the ∼350 base pair enhancer with the non-risk (A) or risk (G) allele of rs58905141 **(A–C)** or the non-risk (T) or risk (G) allele of rs10499197 **(D–F). (G–I)** Dual luciferase assay using Jurkat **(G)**, EBV B **(H)**, or THP-1 **(I)** cells transiently transfected with empty pGL4.23 or pGL4.23 containing the 747 base pair enhancer with the combined non-risk (A,T) or risk alleles (G,G) of rs58905141 and rs10499197. In all experiments, cells were stimulated without or with PMA/Ionomycin for 2 h. Relative luciferase units were calculated as a normalized ratio of Firefly to Renilla; Student’s t-test, n > 3; *p < 0.05; **p < 0.01. (Figure Abbreviations: V, pGL4.23 vector-only; NR, pGL4.23 plus non-risk allele; R, pGL4.23 plus risk allele; P/I, PMA/Ionomycin).

SNP rs58905141 did not exhibit enhancer activation, allelic specificity, or stimulation dependence when expressed in Jurkat or EBV B cells (Figures 2A,B). In THP-1 cells, this sequence demonstrated a two-fold increase in enhancer activity that was lost following stimulation, but no allele-specificity (Figure 2C). In contrast, rs10499197 exhibited enhancer activation in all three immune cell types with THP-1 demonstrating a ten-fold increase (Figures 2D–F). An allelic effect for rs10499197, however, was only detected in EBV B cells, where the risk allele significantly increased luciferase activity compared to the non-risk allele (Figure 2E). When the non-risk or risk alleles of rs58905141 and rs10499197 were cloned in combination, the enhancer activity was reduced in Jurkat and THP-1 cells compared to rs10499197 alone (Figures 2G,I). In contrast, EBV B cells retained similar levels of enhancer activation and the allele-specific effect in the cloned combination as observed with the rs10499197 risk allele alone (Figure 2H). These results demonstrate that the sequence surrounding rs10499197 functions as an enhancer in all 3 cell types, but the risk allele-specific increase of enhancer activation is dependent on factors expressed primarily in EBV B cells.

### The enhancer contacts regulatory elements near *TNFAIP3*, *IL20RA*, *IL22RA2* and *IFNGR1* through long-range chromatin-chromatin interactions

**FIGURE 3:**
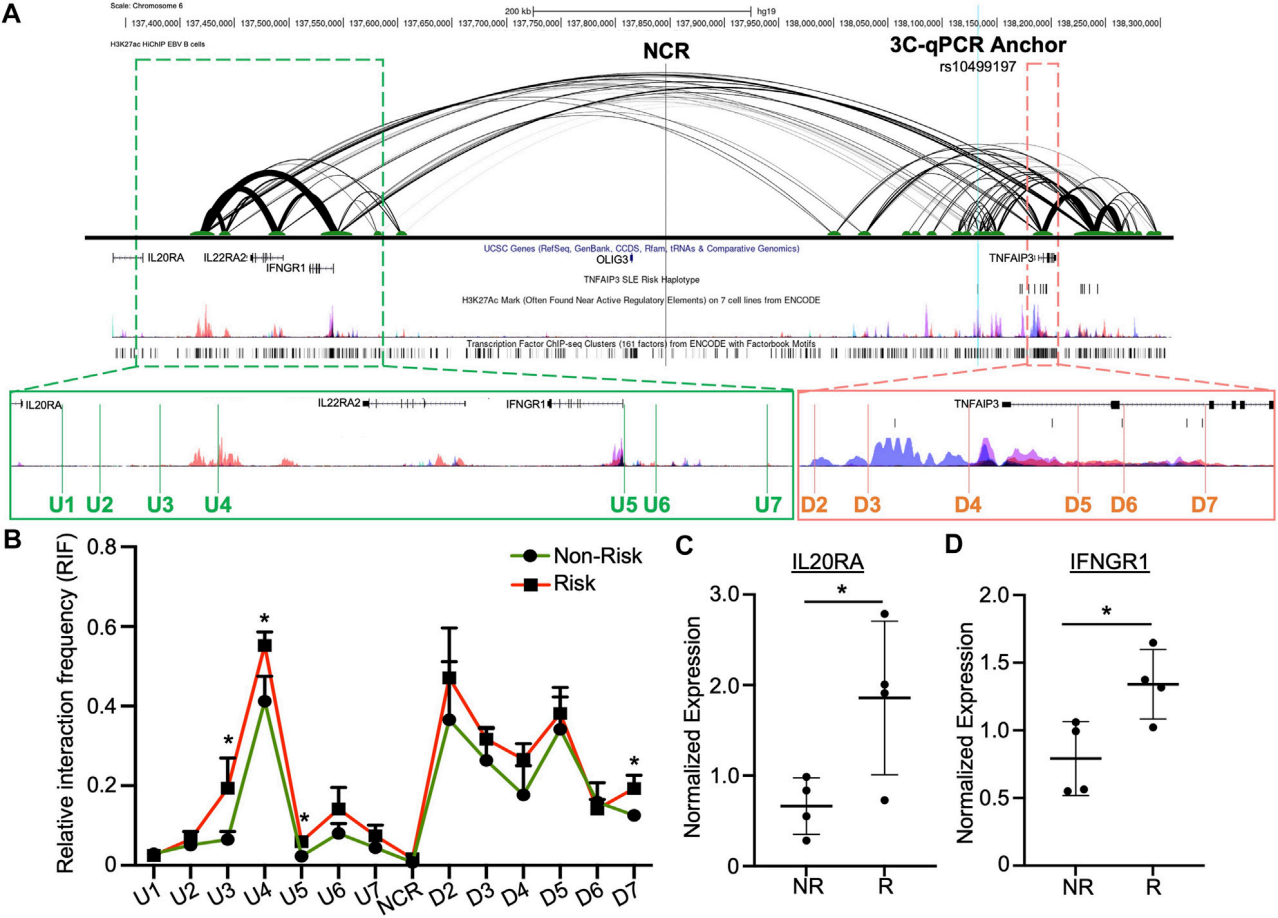
Enhancer upstream of *TNFAIP3* influences distant gene expression through allele-specific chromatin-chromatin looping. **(A)** H3K27ac HiChIP looping interactions within the *TNFAIP3* locus visualized as a two-dimensional diagram. Arc thickness is proportional to the frequency of paired-end tags (6 PET threshold). H3K27ac Peak Track was adapted from the UCSC Genome Browser ENCODE H3K27ac Chromatin marks for GM12878 and six other cell lines. Zoomed outsets depict 3C-qPCR primer locations (green, upstream loop anchors; orange, loop anchors spanning the *TNFAIP3* gene body). **(B)** 3C-qPCR was performed in EBV B cells homozygous for the SLE *TNFAIP3* non-risk or risk haplotype using anchor primers shown in **(A)**. Relative interaction frequency was calculated by normalizing the interaction frequencies to the interaction frequency of the random ligation control and plotted as mean ± SEM, n = 3; Student’s t-test, *p < 0.05. Interactions were considered positive if the relative interaction frequency was greater than the interactions between the anchor and the negative control region of the locus ligation control. **(C,D)** Expression of *IL20RA* **(C)** and *IFNGR1* **(D)** was measured in EBV B cell lines carrying the non-risk or risk SLE haplotype using qRT-PCR. Expression was normalized to *GAPDH*; Student’s t-test, n > 4; *p < 0.05. (Figure Abbreviations: NR, *TNFAIP3* non-risk haplotype; R, *TNFAIP3* risk haplotype; RIF, relative interaction frequency; RLC, random ligation control; NCR, negative control region).

HiChIP analysis in EBV B cells revealed that this enhancer forms multiple H3K27ac-mediated chromatin-chromatin interactions with the downstream *TNFAIP3* gene body and with the upstream regulatory regions of *IL20RA*, *IL22RA2*, and *IFNGR1* (Figure 3A) (Pelikan et al., 2018). We used 3C-qPCR to narrow the locations of the chromatin-chromatin interaction anchors and determine if the *TNFAIP3* SLE risk haplotype tagged by rs10499197 altered the interaction frequencies. We designated the enhancer as the anchor region, then designed PCR primers that were distributed across the genomic sequence between the upstream promoter regions of *IL20RA* and *IFNGR1* to the downstream *TNFAIP3* gene body (Figure 3A; Supplementary Table S3).

In EBV B cells carrying the *TNFAIP3* SLE non-risk or risk haplotype, we observed strong interactions between this enhancer and regulatory elements near *IL20RA* and *IFNGR1*, as well as the promoter region and enhancer elements in the first and second introns of *TNFAIP3* (Figure 3B). Presence of the *TNFAIP3* SLE risk haplotype in EBV B cells significantly increased the interaction frequency between this enhancer and three primers (U3, U4, U5) in the regulatory elements near *IL20RA* and *IFNGR1*, as well as one primer in the second intron of *TNFAIP3* (D7) (Figure 3B). These results suggest that this enhancer may regulate the expression of *IL20RA*, *IL22RA2*, *IFNGR1* and *TNFAIP3* in specific immune cell types, specifically cells of B lymphoid lineage, through long-range chromatin looping. In support, qRT-PCR analysis showed significant increases in the expression of *IL20RA* and *IFNGR1* expression in EBV B cells homozygous for the *TNFAIP3* SLE risk haplotype compared to non-risk haplotype (Figures 3C,D). Expression of *IL22RA2* was not detected in our EBV B cell lines.

### RelA/p65 and CEBPB are integral members of the nuclear protein complex associated with the enhancer

Published ENCODE ChIP-seq data visualized in the UCSC Genome Browser revealed two binding motifs of interest: CCAAT/enhancer-binding protein beta (CEBPB) ∼70 bp upstream and RelA/p65 ∼160 bp downstream of rs10499197 (Supplementary Figure S4). Given that *TNFAIP3* upregulation is an important negative feedback mechanism for NFκB responses, and that the NFκB subunit, RelA/p65, reportedly regulates *TNFAIP3* expression (Wang et al., 2013; Wang et al., 2016), we rationalized that the RelA/p65 core binding motif may contribute to the allele-specific enhancer activity observed using the luciferase assay. Similarly, the p38-responsive CEBPB was previously reported to regulate *TNFAIP3* expression in response to inflammatory stimuli (Lai et al., 2013) and has been repeatedly shown to physically interact with RelA/p65 in other models (Xia et al., 1997; Weber et al., 2003; Khanjani et al., 2011). Consistently, we found that CEBPB co-immunoprecipitated with RelA/p65 in EBV B cell lysates (Supplementary Figure S5A).

**FIGURE 4:**
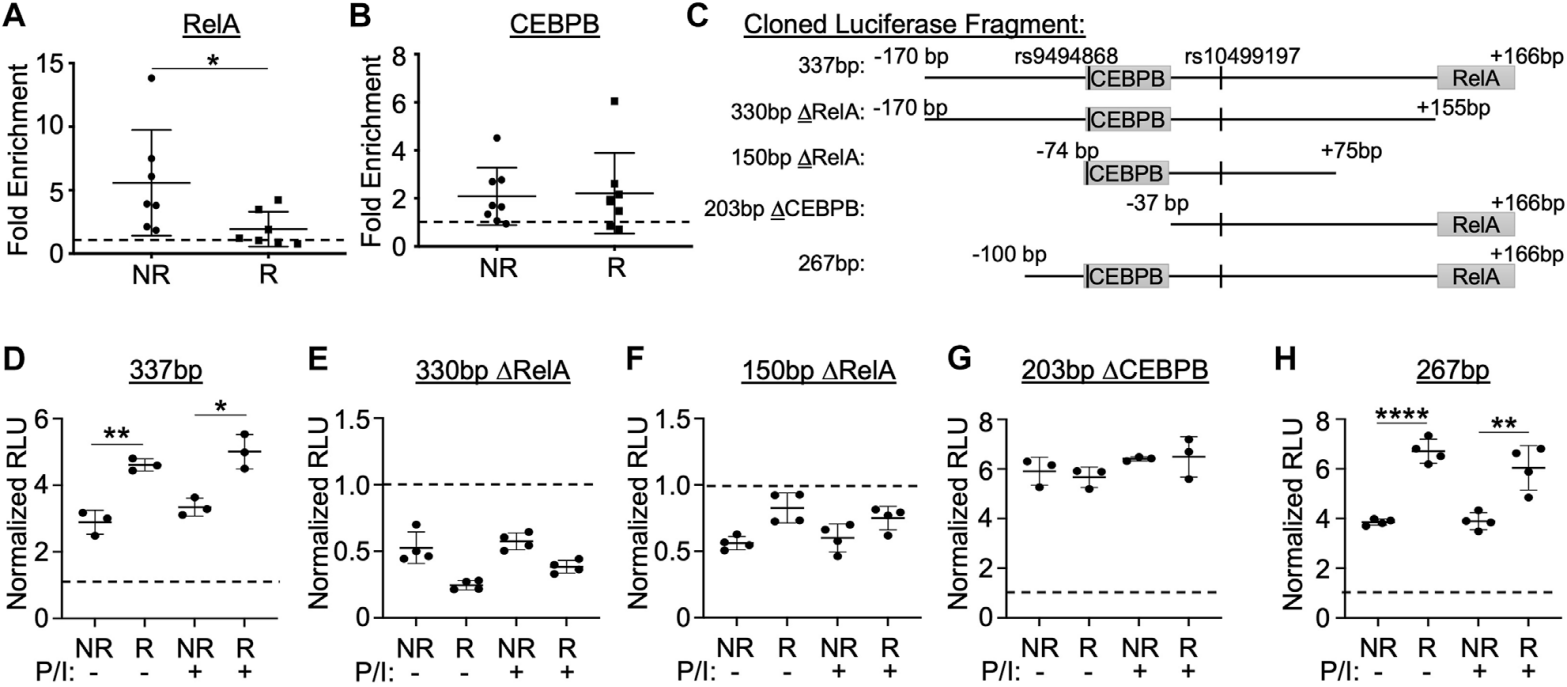
Enhancer function and allele-specific activity is dependent on sequences flanking the RelA/p65 and CEBPB binding motifs. **(A,B)** ChIP-qPCR performed using EBV B cell lines homozygous for the non-risk or risk SLE *TNFAIP3* haplotype (n = 7 cell lines per genotype) and antibodies specific against RelA/p65 **(A)**, CEBPB **(B)**, or rabbit IgG isotype control **(A,B)**. qPCR reported as fold enrichment over isotype control (dotted line). Statistical comparisons were performed using Student’s t-test; *p < 0.05 (Figure Abbreviations: NR, *TNFAIP3* non-risk haplotype; R, *TNFAIP3* risk haplotype). **(C)** Schematic diagram of the truncated regions flanking rs10499197 that were cloned into the pGL4.23 luciferase vector. **(D–H)** Dual luciferase assay using EBV B cells transiently transfected with empty pGL4.23 or pGL4.23 containing the indicated truncated regions in **(C)**. Cells were stimulated without or with PMA/Ionomycin for 2 h. Relative luciferase units were calculated as a normalized ratio of Firefly to Renilla; Student’s t-test, n > 3; *p < 0.05; **p < 0.01; ****p < 0.001. (Figure Abbreviations: V, pGL4.23 vector-only; NR, pGL4.23 with non-risk allele; R, pGL4.23 with risk allele; P/I, PMA/Ionomycin).

Next, we performed EMSA-SS assays using anti-RelA/p65 antibody in combination with the radiolabeled non-risk and risk probes for rs10499197 (Supplementary Figures S5B,C). We observed a loss in nuclear factor binding to the probe in the presence of anti-p65 antibody, suggesting that the anti-p65 antibody blocks p65/RelA binding to the enhancer sequence. To investigate whether RelA/p65 or CEBPB exhibited allele-specific binding to the enhancer region in native chromatin architecture, ChIP-qPCR was performed in EBV B cells homozygous for the *TNFAIP3* SLE risk or non-risk haplotype using anti-p65 or anti-CEBPB antibodies and primers that flanked either the RelA/p65 or CEBPB binding motifs, respectively (Supplementary Table S4). We observed a significant loss of amplicon enrichment for the RelA/p65 motif in cells homozygous for the risk haplotype (Figure 4A) but did not observe any significant difference in amplicon enrichment for the CEBPB motif (Figure 4B).

To further explore the functional importance of the RelA/p65 and CEBPB binding motifs, we cloned several truncated sequences that omitted either the RelA/p65 or CEBPB binding motifs flanking the non-risk or risk alleles of rs10499197 into the minimal promoter luciferase vector (Figure 4C). When compared to the 337bp construct containing both motifs (Figure 4D), loss of the RelA/p65 binding motif (330bp ΔRelA or 150bp ΔRelA) resulted in complete loss of enhancer activity when expressed in EBV B cells (Figures 4E,F), suggesting that RelA/p65 binding is necessary for enhancer activation. In contrast, loss of only the CEBPB binding motif (203bp ΔCEBPB) eliminated the allele-specific differences in luciferase expression (Figure 4G). This allele-specific effect was rescued by a luciferase construct that omitted the first N-terminal 70bp from the 337bp construct (267bp) and retained both the CEBPB and the RelA/p65 motifs (Figure 4H). These results suggest that enhancer function and allele-specific activity is dependent on sequences flanking the RelA/p65 and CEBPB binding sites, respectively, in cells of B lymphoid lineage.

### SNP rs9494868 alters the *in vitro* binding affinities and enhancer activity, but not chromatin-chromatin looping to this enhancer

**FIGURE 5:**
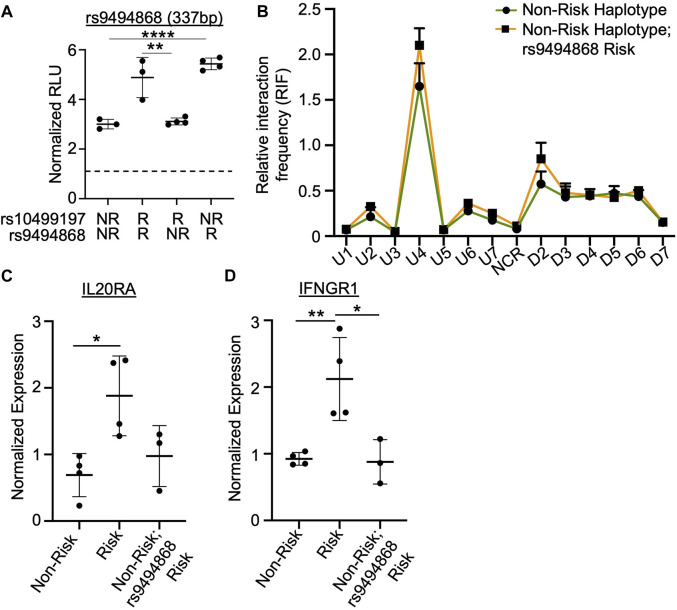
SNP rs9494868 alters the *in vitro* binding affinities and enhancer activity, but not chromatin-chromatin looping to this enhancer. **(A)** Dual luciferase assay using EBV B cells transiently transfected with empty pGL4.23 or pGL4.23 containing the 337bp construct with the indicated non-risk (T) or risk (G) allele of rs10499197 and non-risk (T) or risk (G) allele of rs9494868. Relative luciferase units were calculated as a normalized ratio of Firefly to Renilla; Student’s t-test, n > 3; ****p < 0.001 (Figure Abbreviations: V, pGL4.23 vector-only; NR, pGL4.23 with non-risk allele; R, pGL4.23 with risk allele). **(B)** 3C-qPCR was performed in EBV B cells homozygous for the SLE *TNFAIP3* non-risk haplotype and either the non-risk (T) or risk (G) allele of rs9494868 using primers shown in Figure 3A. Relative interaction frequency was calculated by normalizing the interaction frequencies to the interaction frequency of the random ligation control and plotted as mean ± SEM, n = 3; Student’s t-test, *p < 0.05. Interactions were considered positive if the relative interaction frequency was greater than the interactions between the anchor and the negative control region of the locus ligation control. **(C,D)** Expression of *IL20RA* **(C)** and *IFNGR1* **(D)** were measured in EBV B cells for the SLE *TNFAIP3* non-risk haplotype, SLE *TNFAIP3* risk haplotype, or SLE *TNFAIP3* non-risk haplotype and the risk allele (G) of rs9494868 using qRT-PCR. Expression was normalized to *GAPDH*; Student’s t-test, n > 3; *p < 0.05; **p < 0.01.

Sanger sequencing of the luciferase constructs derived from EBV B cell lines homozygous for the SLE risk allele of rs10499197 revealed a SNP, rs9494868 (T for non-risk; G for risk), positioned at the most 5′ base pair of the CEBPB binding motif. SNP rs9494868 is in strong LD with rs10499197 (*D’* = 0.999), but the two are not strongly correlated (*r* ^*2*^ = 0.074). Site-directed mutagenesis and subsequent luciferase assays were performed to determine if the rs9494868 risk allele also influenced the observed increase in enhancer activity by luciferase assay. In the context of the rs10499197 risk allele, the non-risk allele of rs9494868 reduced the allele-specific increase in enhancer activity in EBV B cells (Figure 5A). However, in the context of the rs10499197 non-risk allele, the risk allele of rs9494868 increased enhancer activity (Figure 5A), suggesting that rs9494868, rather than the rs10499197, may regulate the observed allelic effect on enhancer activity in our EBV B cell luciferase assay.

Due to differences in the risk allele frequencies of rs10499197 and rs9494868 (3 vs. 23%, respectively) in cohorts of European ancestry, an individual can have the *TNFAIP3* non-risk haplotype but the risk allele of rs9494868. Therefore, we performed 3C-qPCR using EBV B cells that were homozygous for the *TNFAIP3* SLE non-risk haplotype with or without the risk allele of rs9494868 to determine if rs9494868 influenced the haplotype-specific changes in the observed chromatin-chromatin interactions at this locus. Interestingly, presence of the rs9494868 risk allele did not significantly alter interaction frequencies observed in EBV B cells carrying the *TNFAIP3* SLE non-risk haplotype (Figure 5B). Further, the rs9494868 risk allele alone wasn’t sufficient to alter the expression of *IL20RA* or *IFNGR1* (Figures 5C,D). These results suggest that the combined influence of the rs9494868 risk allele and other variants on the SLE risk haplotype increase the enhancer activity necessary for haplotype-specific expression of *IL20RA* and *IFNGR1*. Given the rarity of the SLE risk haplotype with the non-risk allele of rs9494868 in our cohort, we cannot definitively demonstrate the inverse of this experiment in the natural genomic context; however, the luciferase assays strongly suggest that the risk allele of rs9494868 drives the allele-specific enhancer activity.

## Discussion

This study aimed to functionally characterize a predicted enhancer located ∼55 kb 5′ of the *TNFAIP3* promoter. Although this enhancer is included in several autoimmune disease haplotypes spanning the *TNFAIP3* locus, including the SLE risk haplotype studied herein, and has common SNPs, rs10499197 and rs58905141, shown to alter chromatin dynamics and enhancer activity, the functional significance and allelic effects on gene expression has remained unclear. By leveraging patient-derived EBV B cell lines that were homozygous for either the non-risk or risk *TNFAIP3* SLE risk haplotype tagged by rs10499197, we uncovered a complex chromatin regulatory network that spans ∼1M bp from the promoter region of *IL20RA* to the 3’ untranslated region of *TNFAIP3* (Adrianto et al., 2011; Wang et al., 2013; Wang et al., 2016).

Dissection of the enhancer and the allelic effects of rs10499197 and rs9494868 using luciferase assays demonstrated a co-dependency for sequences that bind CEBPB and RelA. Together, the two binding motifs increase risk allele-specific expression of *IL20RA* and *IFNGR1*. While other independent risk haplotypes have been associated with long-range looping and allele-specific modulation of *IL20RA* and *IFNGR1* gene expression (McGovern et al., 2016), our results are the first to show participation of the ∼109 kb SLE risk haplotype in this activity. Since the SLE risk haplotype has been shown to suppress *TNFAIP3* expression, we expected the enhancer element tagged by rs10499197 to exert its function similarly as we previously described for the TT>A enhancer element (Adrianto et al., 2011; Wang et al., 2013; Wang et al., 2016). However, the underlying functional mechanism appears to be more nuanced to accommodate hypermorphic expression of *IL20RA* and *IFNGR1* and hypomorphic expression of *TNFAIP3*. For example, the enhancer effect is dependent on sequences flanking the RelA/p65 binding motif (Figure 4E) but does not appear to be dependent on RelA/p65 based on our ChIP-qPCR experiments that showed a loss of RelA/p65 binding with the risk haplotype (Figure 4A). At the same time, the allele-specific effect is dependent on sequences flanking rs9494868 and the CEBPB binding motif (Figure 4G and Figure 5A), but CEBPB binding is not altered in an allele-specific manner by ChIP-qPCR (Figure 4B). It is likely that other uncharacterized transcription modulators are involved in coordinating these effects.

Our data from EBV B cell lines that carry the risk allele of rs9494868 but do not carry the SLE risk haplotype suggest that, for the hypermorphic expression of the *IL20RA* and *IFNGR1*, increased looping to the enhancer regions flanking these genes is required along with increased enhancer activity promoted by the SLE risk alleles. Due to the rarity of EBV B cells that carry the SLE risk haplotype without the risk allele of rs9494868, we could not assess the possibility that the allelic effect would be lost while the enhancer effect was preserved. Further work using engineered cell lines could potentially answer this outstanding question. As for the impact of this enhancer on hypomorphic *TNFAIP3* expression, our demonstrations of the enhancer looping to promoter/enhancer elements within the *TNFAIP3* gene body and the allele-specific loss of RelA/p65 binding could support a role in *TNFAIP3* suppression, which is characteristic of other variants carried on the SLE risk haplotype (Adrianto et al., 2011; Wang et al., 2013; Wang et al., 2016; Sokhi et al., 2018; Ray et al., 2020). In addition, our serendipitous discovery of rs9494868 demonstrates the importance of considering both D’ and *r* ^2^ LD parameters when defining risk haplotypes for functional characterization of disease-associated risk SNPs.

Our study has limitations that should be considered in the interpretation of our results. We used a combination of *in vitro* and *in vivo* (cellular) analyses to explore variant function. The *in vitro* assays are straight forward for assessing allele-specific effects but, since they do not represent the true genomic context, results can conflict with orthogonal studies performed in intact cells. We also used EBV B cell lines that carried specific combinations of risk haplotypes. While EBV B lines are a valuable resource for genomic studies in many ways, each has their own genomic personality due to endogenous genetic variation and alterations incurred from transformation, which adds heterogeneity to our assays.

The *TNFAIP3* locus is highly impacted by a diversity of risk haplotypes across multiple autoimmune and immune-mediated diseases. Our results support previous studies that suggest this locus should be expanded into a super-locus that includes *IL20RA* and *IFNGR1*. While the hypomorphic expression of *TNFAIP3* has been described in several settings and leads to increased inflammation, hypermorphic expression of *IL20RA* and *IFNGR1* could exacerbate the pro-inflammatory state. *IL20RA* encodes the alpha subunit of the Interleukin-20 (IL-20) receptor. The IL-20 signaling axis influences the crosstalk between lymphocytes and epithelial cells, including facilitating the infiltration of immune cells to the kidneys in murine lupus nephritis models (Wu et al., 2022). IL-20 signaling also activates proinflammatory responses and reactive oxygen species production that contribute to renal tissue damage. *IFNGR1* encodes the IFNγ receptor subunit 1, expressed on the surface of B cells. Elevated expression of *IFNGR1* and subsequent IFNγ signaling activation promotes the loss of self-tolerance and production of autoantibodies in murine lupus models (Bacalao and Satterthwaite, 2021). Our study suggests that the *TNFAIP3* risk haplotype associated with SLE and other autoimmune diseases (e.g. rheumatoid arthritis, psoriasis, multiple sclerosis, inflammatory skin disorder, inflammatory bowel disease, celiac disease, and Sjögren’s disease (Graham et al., 2008; Trynka et al., 2009; Dieudé et al., 2010; Shimane et al., 2010; Musone et al., 2011; Koumakis et al., 2012; Zhou et al., 2015; Xu et al., 2019; Khatri et al., 2022) not only results in the loss of an important suppressor of immune stimulation but may also concomitantly activate the immune system through IL-20 and IFNγ signaling pathways in specific cellular contexts.

## Data Availability

The HiChIP datasets presented in this study are available from the NCBI GEO database (https://www.ncbi.nlm.nih.gov/geo/) with project ID: GSE116193. All other relevant data are within the manuscript and supporting information files.
