# Supplementary material for: Systemic lupus erythematosus variants modulate the function of an enhancer upstream of TNFAIP3
Source: Front Genet. 2022 Sep 19;13:1011965. doi: 10.3389/fgene.2022.1011965 (PMC9527318; doi:10.3389/fgene.2022.1011965)
Supplement: Supplementary file 1 [file DataSheet1.pdf]

Supplementary Material

Systemic lupus erythematosus variants modulate the function of an enhancer upstream of *TNFAIP3*

Satish Pasula[1], Jaanam Gopalakrishnan[1,2], Yao Fu[1], Kandice L. Tessneer[1], Mandi M. Wiley[1], Richard C. Pelikan[1], Jennifer A. Kelly[1], and Patrick M. Gaffney[1,2*]

[1]Genes and Human Disease Research Program, Oklahoma Medical Research Foundation, Oklahoma City, Oklahoma, United States of America

[2]Department of Pathology, University of Oklahoma Health Sciences Center, Oklahoma City, Oklahoma, United States of America

*** Correspondence:**
Patrick M. Gaffney, M.D., Chair
patrick-gaffney@omrf.org

Keywords: systemic lupus erythematosus, TNFAIP3, IL20RA, IFNGR1, functional genetics

Conflict of Interest: The authors declare that the research was conducted in the absence of any commercial or financial relationships that could be construed as a potential conflict of interest.

Funding: Research reported in this publication was supported by the National Institute of General Medical Sciences, the National Institute of Arthritis and Musculoskeletal and Skin Diseases, and the National Institutes of Allergy and Infectious Diseases of the National Institutes of Health (NIH) grants: R01AR063124, R01AR073606, R01AR056360, P30GM110766, U19AI082714, R01AI156724; as well as the Presbyterian Health Foundation. The ORDRCC (source of EBV B cell lines) is supported by the National Institutes of Health grants U54GM104938, P30AR073750, P30GM103510 and P20RR020143. The content of this publication is solely the responsibility of the authors and does not represent the official views of the funding agencies.

1. Supplementary Figures and Tables

1.1 Supplementary Figures

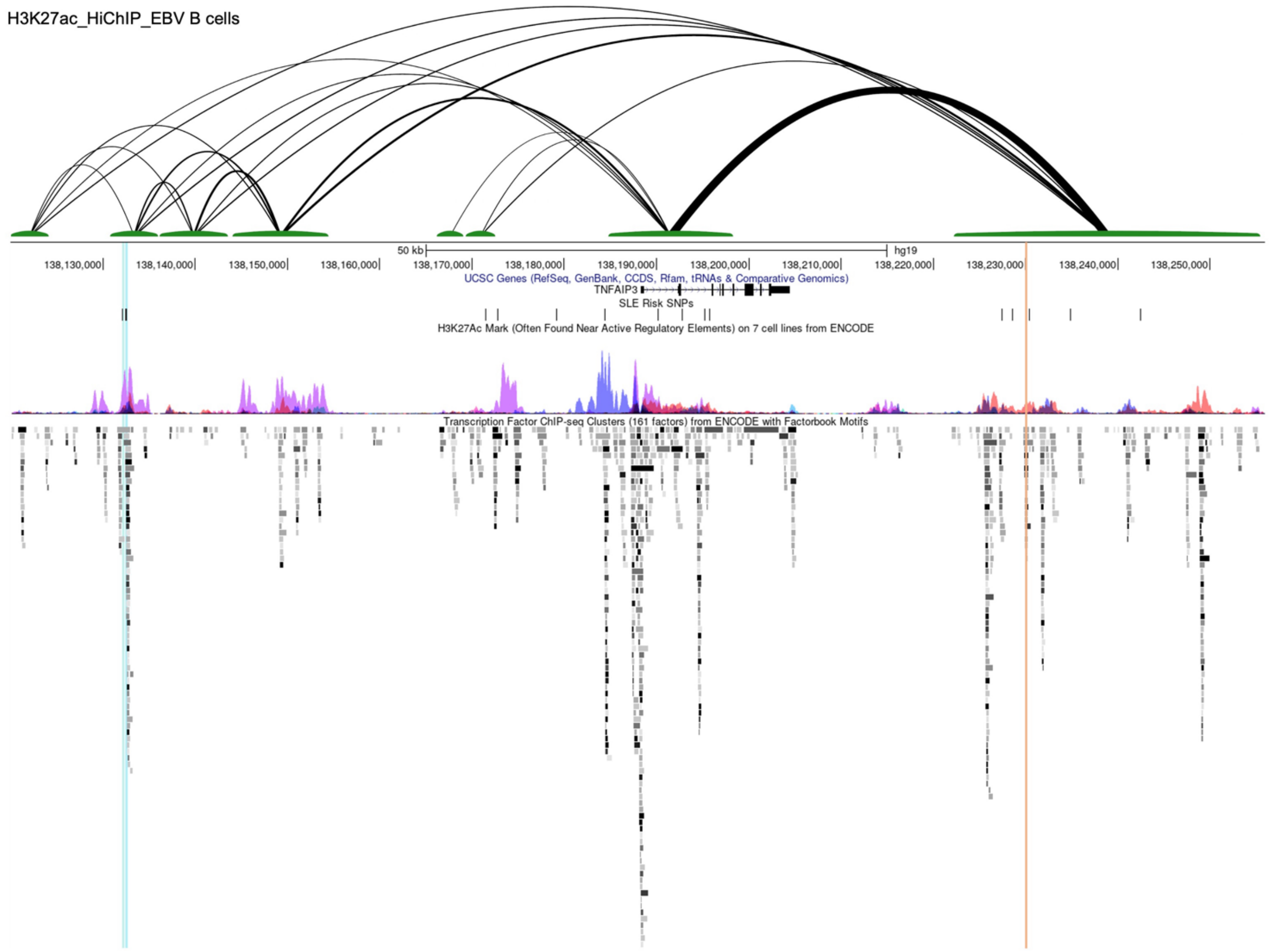

Supplementary Figure S1. SLE risk variants, rs10499197, rs58905141 are positioned in an enhancer with complex 3D chromatin structure ~55 kb upstream of *TNFAIP3*. Three-dimensional organization of H3K27ac HiChIP looping interactions at the *TNFAIP3* locus is visualized as a two-dimensional looping diagram. HiChIP revealed interactions between the *TNFAIP3* gene body and the well-characterized TT>A enhancer (rs14314164, rs200820567; orange line) downstream of *TNFAIP3*, as well as an enhancer at position 138.14 (~55 kb upstream of the *TNFAIP3* promoter) that overlaps two SLE risk alleles, rs58905141 and rs10499197 (blue lines). Identified enhancers are consistent with ENCODE H3K27ac ChIPseq peaks for GM12878 EBV B, K562 lymphoblastoid, and H1-hESC cell lines, and transcription factor ChIP-seq clusters from ENCODE.

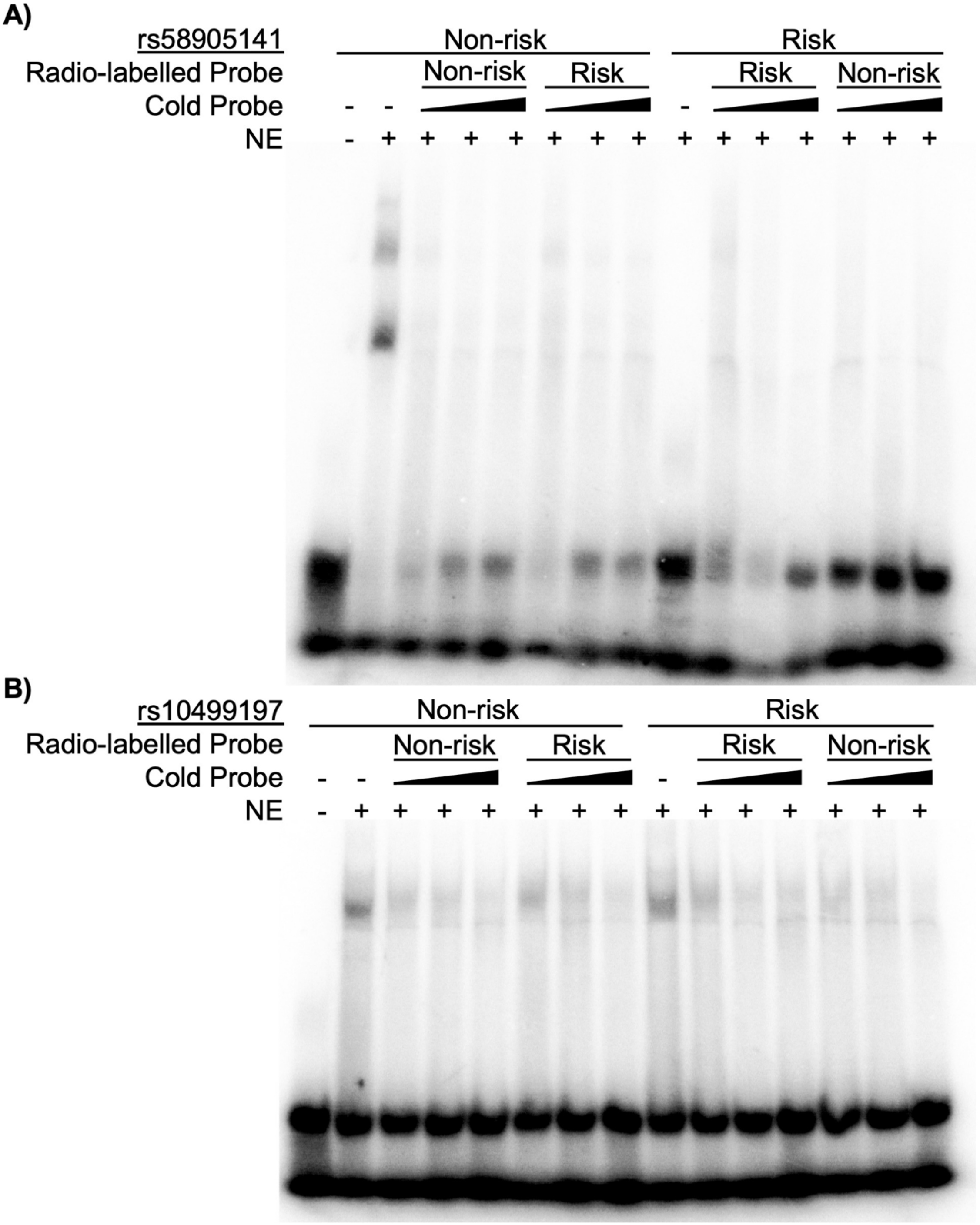

Supplementary Figure S2. Cold-probe competition assay demonstrates nuclear protein binding specificity. Cold-probe competition EMSAs using nuclear extracts from THP-1 cells and radiolabeled probes of the non-risk (A) or risk (G) allele of rs58905141 (**A**) or radiolabeled probes of the non-risk (T) or risk (G) allele of rs10499197 (**B**). Cold probes were used at 10X, 50X, or 100X concentration of the cold probe; indicated by black triangle. Images are representative of n>2. (Abbreviations: NE, nuclear extract).

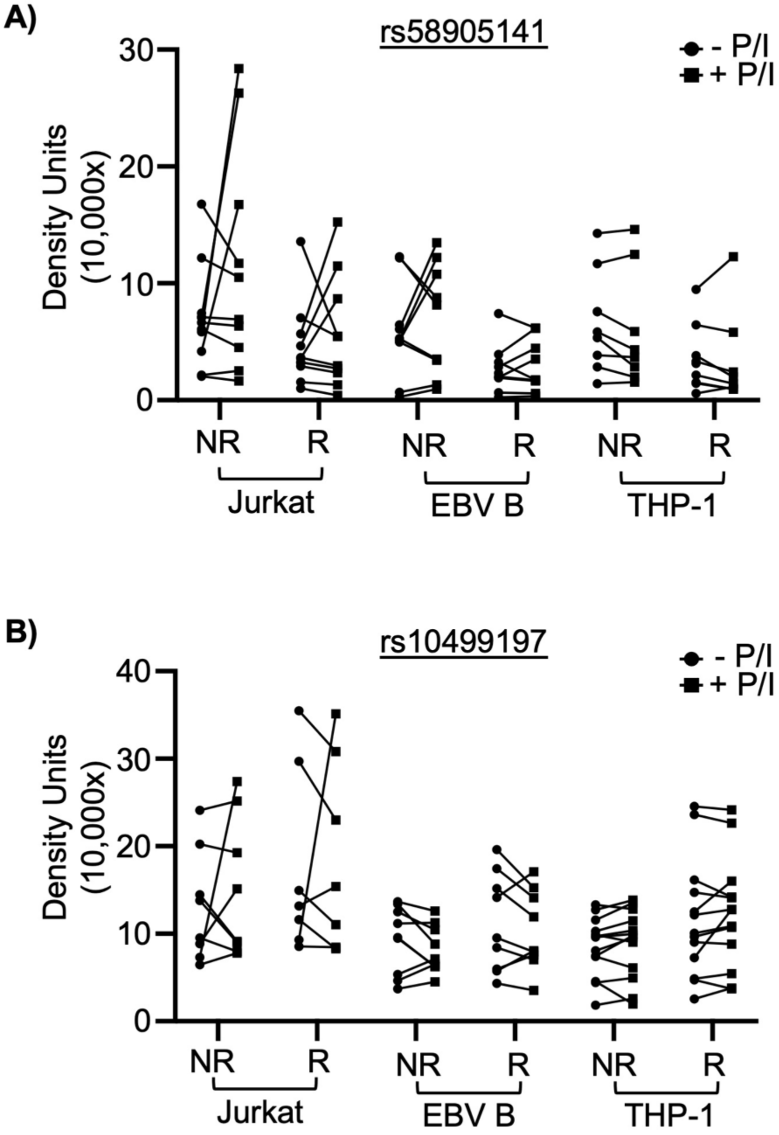

Supplementary Figure S3. ***TNFAIP3*** **rs58905141 and rs10499197 alleles are not stimuli-responsive.** EMSA using nuclear extracts from Jurkat, EBV B, and THP-1 cells stimulated with or without PMA/Ionomycin for 2 h. Radiolabeled probes of the non-risk (A) or risk (G) allele of rs58905141 (**A**) or non-risk (T) or risk (G) allele of rs10499197 (**B**) were incubated with nuclear extracts as indicated. Representative images shown in Figure 1A and 1C; n>6. Densitometry was used to quantify nuclear protein-bound DNA probe; paired t-test: not significant. (Figure Abbreviations: NE, nuclear extract; NR, non-risk allele; R, risk allele; P/I, PMA/Ionomycin).

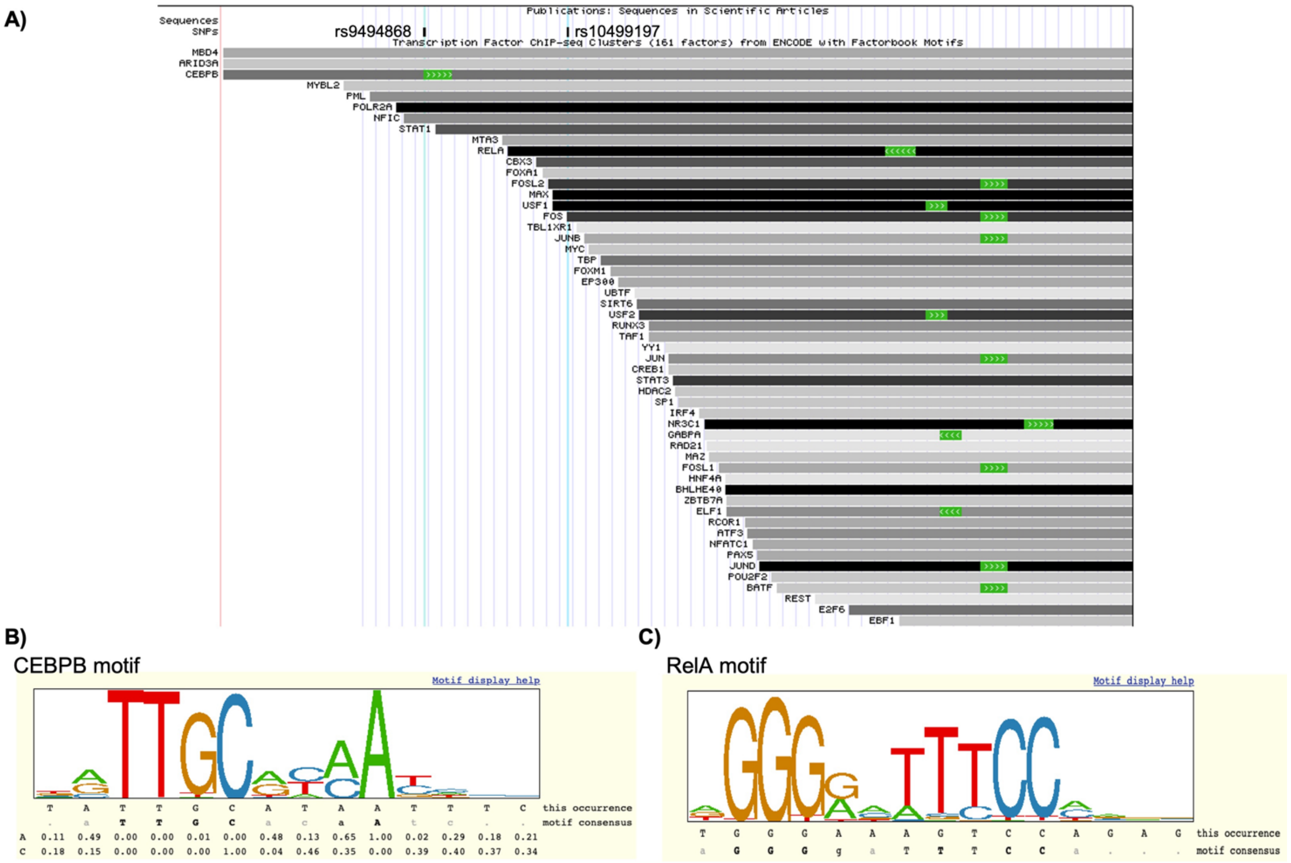

Supplementary Figure S4. RelA/p65 and CEBPB are integral members of the nuclear protein complex associated with the enhancer. (A) ChIP-seq data visualized in the UCSC Genome Browser revealed two binding motifs of interest: CCAAT/enhancer-binding protein beta (CEBPB) ~70 bp upstream and RelA/p65 ~160 bp downstream of rs1049917. Blue lines indicate the positioning of rs9494868 and rs10499197 variants. (**B-C**) Encode predicted CEBPB (**B**) and RelA (**C**) motif regions aligned with respective motif consensus.

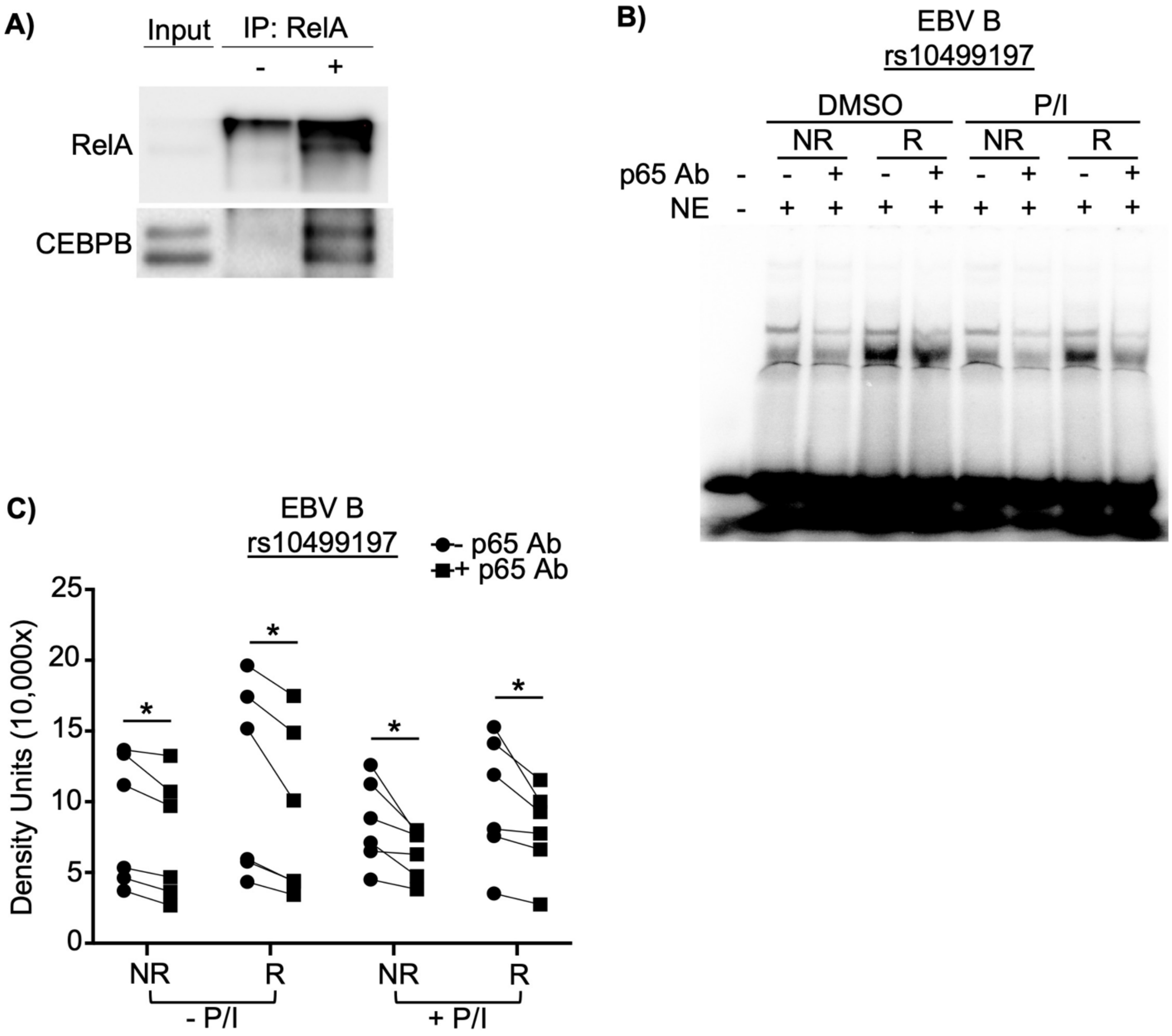

Supplementary Figure S5. ***TNFAIP3*** **rs10499197 exhibits allele-specific RelA/p65 binding.** (**A**) Co-immunoprecipitation of CEBPB by RelA in cell extracts from quiescent EBV B cells. Images are representative immunoblots of n=3. (**B-C**) EMSA Supershift assay using nuclear extracts from EBV B cells stimulated with or without PMA/Ionomycin for 2 h. Radiolabeled probes with the non-risk (T) or risk (G) allele of rs10499197 were incubated with nuclear extracts supplemented with or without anti-p65-specific antibody as indicated. (**B**) Image is representative of n>5. (**C**) Densitometry was used to quantify nuclear protein-bound DNA probe; paired t-test: *$p<0.05$. (Figure Abbreviations: Ab, antibody; IP, Immunoprecipitation; NE, nuclear extract; NR, non-risk allele; R, risk allele; P/I, PMA/Ionomycin)

1.2 Supplementary Tables

Supplementary Table S1. EMSA Oligonucleotide Probe Sequences

Radiolabeled Probes (Non-Risk or Risk Allele)	Forward Probe	Reverse Probe
rs58905141 Non-risk (A)	ATCCATCATGTTCTTCCTTGA AACAACTCTTAAACTCTTAC	GTAAGAGTTTAAGAGTTGTTTCAAG GAAGAACATGATGGAT
rs58905141 Risk (G)	ATCCATCATGTTCTTCCTTGG AACAACTCTTAAACTCTTAC	GTAAGAGTTTAAGAGTTGTTCCAAG GAAGAACATGATGGAT
rs10499197 Non-risk (T)	CTGAGACCTACTTACTTAGCT TCCAAATATGGTAGTAATTA	TAATTACTACCATATTTGGAAGCTA AGTAAGTAGGTCTCAG
rs10499197 Risk (G)	CTGAGACCTACTTACTTAGCG TCCAAATATGGTAGTAATTA	TAATTACTACCATATTTGGACGCTA AGTAAGTAGGTCTCAG

Supplementary Table S2. Dual Luciferase Assay Primer Sequences

Cloning Primers	Forward Primer	Reverse Primer
rs58905141 (383bp)	ACTGGTACCGATAACTGATTT GGACTTTGCT	AGTCTCGAGCAGAGGTTTAGG GAGAGAAAT
rs10499197 (337bp)	AGCAGGTACCCCACACTGACA AAGCCAATGT	TCTACTCGAGGTCCAGAGTAG ACTGAACGTACT
330bp ΔRelA	AGCAGGTACCCCACACTGACA AAGCCAATGT	AGTCTCGAGACTGAACGTACT TTTTAAGAAAAGGA
150bp ΔRelA	ACTGGTACCTGATTATTGCATA ATTTCATCCT	AGTCTCGAGTTTGATAGTTCT GTGCAAGGCA
203bp ΔCEBPB	AGTGGTACCTGCTCTTTATTTA ATGACTGAGA	TCTACTCGAGGTCCAGAGTAG ACTGAACGTACT
267bp ΔCEBPB	AGTGGTACCTAATTTATTATTT AACCAAAG	TCTACTCGAGGTCCAGAGTAG ACTGAACGTACT
**Site-Directed Mutagenesis Primers**	**Forward Primer**	**Reverse Primer**
rs10499197 (G>T)	CCTACTTACTTAGCTTCCAAAT ATGGTAG	CTACCATATTTGGAAGCTAAG TAAGTAGG
rs9494868 (G>T)	CCAAAGCCAGTGATTATTGCA TAATTTCATCC	GGATGAAATTATGCAATAATC ACTGGCTTTGG

Supplementary Table S3. 3C-qPCR Oligonucleotide Primer Sequences

Primer	Sequence	Start Position	End Position	Cutting Site
Anchor	AGAGGAAAGGACAGACTTGAG	138132791	138132811	138132883
U1	CAGGGCTTGACAAGACATTTCTC	137377272	137377295	137377334
U2	TCCGAAGAGCTTTGTTTGTGG	137381301	137381322	137381475
U3	TGCTGCCCAGACATAGGAAA	137406792	137406812	137407040
U4	TGCCATTCTAGCCCTTCCAG	137423110	137423130	137423210
U5	GCCAAGTGCCTATTACTCCAT	137544290	137544311	137544439
U6	TCTACTGGTGCACATTTCCT	137555115	137555135	137555184
U7	CAAGGCAAGGTGGTGGTTTT	137583168	137583188	137583223
D1	GTGACAGAGTGAGACCTTGT	138180102	138180121	138180192
D2	TAACCCAACACTTAGGCTCA	138182379	138182399	138182573
D3	TGGGTTTCTCCATTCAGTCC	138184563	138184582	138184707
D4	AGACTGGTCATTATGGGCTT	138186764	138186783	138186854
D5	GTTTTGTCCTCAGTTTCGGG	138192529	138192548	138192635
D6	TAGGTTCTATTGCCTATGCCC	138193187	138193207	138193357
D7	TTGTAGAGTGATGTCAGAATGA	138196743	138196764	138196796
NCR	ACAGGCAGTGGTATGTTGGA	137833999	137834018	137834160
RLC	CTTGACACTGATCCCTCCAAT	138126775	138126795	138126865

Supplementary Table S4. ChIP-qPCR Primer Sequences

	Forward Primer	Reverse Primer
RelA/p65	TAACCTTGTGACACAGCCCT	GGTCAGCTAACAGAGCAGGAA
CEBPB	GTTCTGCTTCACAATTCGGCA	AAGAGCAGCATTCTTGATTGGAA
